# Modelling Nitrogen Excretion in Dairy Cows: An Application to Farms in the Po Valley (Italy)

**DOI:** 10.3390/ani16020294

**Published:** 2026-01-17

**Authors:** Valentina Caprarulo, Elena Scaglia, Anna Simonetto, Giulia Ferronato, Valeria Sergi, Laura Giagnoni, Gianni Gilioli

**Affiliations:** 1 Department of Civil, Environmental, Architectural Engineering and Mathematics, Università di Brescia, 25121 Brescia, Italy; elena.scaglia@unibs.it (E.S.); anna.simonetto@unibs.it (A.S.); giulia.ferronato@unibs.it (G.F.); valeria.sergi@unibs.it (V.S.); laura.giagnoni@unibs.it (L.G.); gianni.gilioli@unibs.it (G.G.); 2 Istituto Zooprofilattico Sperimentale della Lombardia e dell’Emilia Romagna “Bruno Ubertini”, 25124 Brescia, Italy

**Keywords:** nitrogen excretion, prediction model, dairy cows, nitrogen balance, nitrates directive compliance

## Abstract

Effective nitrogen management in dairy cow diets is essential for optimising milk production and minimising environmental nitrogen emissions. This study develops a simplified model to estimate nitrogen excretion in dairy farms, distinguishing excretion by animal category (lactating cows, heifers, calves) and organic matrix (faeces, urine), with nitrogen intake as a key input. A comprehensive literature review guided the selection of equations for estimating nitrogen excretion based on dietary nitrogen content, dry matter intake and milk yield. The model was specifically calibrated for Holstein dairy herd in the Po Valley (Italy) context using data collected from ten Lombardy dairy farms over 30 months, focusing on diet composition and nitrogen excretion via faeces, urine, and milk. Validation against established the literature and the Nitrates Directive (91/676/EEC) excretion factors demonstrated the model’s alignment in estimating nitrogen excretion. Within this context, the proposed framework may support nitrogen management at farm level by providing a practical, descriptive tool to explore nitrogen flows and to identify potential areas for improving nutrient efficiency and reducing environmental impacts.

## Simple Summary

Nitrogen excretion from dairy cattle is a major contributor to environmental pollution and is subject to strict regulations in many European regions. Reliable and practical tools are therefore needed to estimate nitrogen excretion under different production conditions and to support farm-level environmental management. In this study, we developed and applied a simplified herd-level model to estimate nitrogen excretion in dairy cows under the production conditions of the Po Valley (Italy). The model relies on laboratory measurements of nitrogen excretion in faeces, urine, milk, and manure for lactating cows, together with literature-based data for other animal categories. The model is conceived as a dynamic tool that can be used by dairy farms to periodically assess their environmental performance and to generate herd-specific nitrogen excretion coefficients. Repeated assessments over time allow deviations from expected reference values to be identified, thereby facilitating the detection of management or nutritional inefficiencies and the definition of targeted mitigation actions. The model can also support compliance with environmental regulations, including those related to nitrate management.

## 1. Introduction

The dairy sector is a cornerstone of global food systems, supplying high-value products such as milk, meat, and a wide range of dairy derivatives. Ruminants hold a unique position in human nutrition because their digestive physiology enables the transformation of fibrous plant material, otherwise indigestible by humans, into highly bioavailable proteins [1]. Despite these nutritional and socioeconomic benefits, dairy farming is closely associated with significant environmental pressures, particularly those related to nitrogen (N) emissions. Nitrogen excreted by livestock is essential for maintaining soil fertility and sustaining agricultural circularity; however, when mismanaged it becomes a major contributor to greenhouse gas emissions and a driver of soil and water degradation [2,3]. Excess reactive nitrogen is widely recognised as a source of soil and water acidification, eutrophication, and biodiversity losses across terrestrial and aquatic ecosystems [4,5,6].

Recent global assessments indicate that total N_2_O emissions reached approximately 17.8 Tg N year^−1^ during the 2010–2019 period, with agricultural activities responsible for nearly three-quarters of anthropogenic emissions [7]. In 2020 alone, direct agricultural emissions were estimated at around 3.9 Tg N year^−1^, confirming agriculture as the dominant anthropogenic source of N_2_O. In Europe, the agricultural sector contributes roughly 11% of total greenhouse gas emissions [8], and in Italy it represents the main source of N_2_O, accounting for 64.8% of national emissions due to fertiliser use and livestock effluent management [9]. Moreover, within the European Union, nitrate pollution of water resources is regulated under the Nitrates Directive (91/676/EEC), which seeks to curb nitrogen losses from soils to groundwater and surface waters.

These regulatory measures are fully aligned with broader environmental commitments, including the Sustainable Development Goals at the global scale and the Green Deal at the European level, and the “Fit for 55” initiative, which aims to achieve a 55% reduction in greenhouse gas emissions by 2030 [10,11]. Within this evolving regulatory and policy framework, improving nitrogen management at both herd and farm levels has therefore emerged as a strategic priority for the dairy sector. In this context, feeding strategies constitute one of the most effective levers for mitigating nitrogen emissions while sustaining productive performance. Ruminants excrete between 60% and 80% of ingested nitrogen via faeces and urine [12], and dietary interventions can significantly influence total N intake and its partitioning between excretory pathways. More recently, observational work on Italian dairy farms has demonstrated that distinct feeding strategy can substantially influence both nitrogen and methane emissions [13], while innovation analyses highlight that nutritional interventions, particularly feed additives, are among the most actively developed approaches for reducing emissions in ruminants [14]. From this perspective, quantifying nitrogen excretion at the farm scale becomes essential for designing and evaluating mitigation strategies tailored to local environmental vulnerabilities, particularly in areas prone to nitrate leaching.

Although direct measurements of nitrogen flows provide accurate and detailed information, their application in commercial dairy farms is limited by high costs, labour requirements and logistical constraints. These limitations have stimulated the development of predictive approaches for estimating nitrogen excretion, including empirical equations, mechanistic models and whole-farm simulation tools [15,16,17,18,19,20,21]. In particular, simplified predictive tools based on routinely collected farm data are considered especially promising for practical implementation as they allow nitrogen excretion to be estimated under commercial farming conditions and support the evaluation of dietary mitigation strategies at herd and farm scale [22]. Nevertheless, each of these approaches presents relevant constraints. Empirical models are often calibrated on specific datasets and subsequently extrapolated beyond their original domain of validity, which may compromise their reliability across different production contexts. Conversely, mechanistic and process-based models require extensive input data and high computational demand, thus remaining largely confined to research applications rather than routine farm advisory use [20,23]. At a broader scale, inventory-oriented approaches typically rely on generic emission factors that fail to account for farm-specific management practices and therefore provide limited support for decision-making at the herd level. However, modelling frameworks of this type may still represent a valuable tool for refining national emission inventories through the development of context-specific nitrogen excretion factors tailored to local production systems [24]. As a result, a clear gap persists between the modelling tools currently available and the need for simple and system-sensitive approaches that can effectively support farmers, technical advisors, and regional authorities—particularly in nitrate-vulnerable areas.

The present study directly addresses this gap by developing a simplified, farm-scale model designed to estimate herd nitrogen excretion within the barn. Unlike existing approaches, the model (i) is calibrated using detailed, real-farm data from Holstein herds fed Total Mixed Rations (TMR) in the Po Valley (Italy); (ii) requires only routinely collected farm inputs, enabling practical application in commercial settings; (iii) captures diet-driven variation in nitrogen excretion, thus bridging the divide between empirical ease-of-use and biological relevance; and (iv) in addition, the model is conceived as a dynamic tool that can be used by farms to periodically evaluate their environmental performance, to generate herd-specific nitrogen excretion coefficients, and to support compliance with environmental regulations, including those related to nitrate management. The availability of repeated assessments over time also allows deviations from expected reference values to be identified, thereby facilitating the diagnosis of management or nutritional inefficiencies and the definition of targeted mitigation actions.

Model calibration and validation were performed using comprehensive datasets collected over two years from ten commercial dairy farms located in the provinces of Brescia and Cremona (Lombardy region, Italy).

## 2. Materials and Methods

The objective of this study was to develop and calibrate a simplified farm-scale model for estimating nitrogen (N) excretion in dairy herds managed under production conditions typical of the Po Valley. The model quantifies nitrogen flows occurring within the barn system boundary, where nitrogen excretion is defined as the daily amount of N eliminated by the animal through faeces (NexF), urine (NexU) and, for lactating cows, milk (NexM). Total nitrogen excretion (NexTot) is expressed as the sum of these components. Nitrogen balance (NB) is defined as the difference between nitrogen intake (NI) and NexTot and corresponds to the quantity of N retained in the body for maintenance, growth and gestation. All variables were expressed on a daily basis (g N cow^−1^ d^−1^), and annual estimates were subsequently converted to kg N head^−1^ year^−1^ to allow comparison with regulatory benchmarks.

The modelling framework was tailored to Italian Holstein cows fed total mixed rations (TMR) and was implemented across five animal categories: pre-weaning calves (0–3 months), post-weaning calves (3–7 months), heifers (7–24 months), dry cows and lactating cows. Because nitrogen metabolism, nutrient requirements and feeding strategies differ markedly among these categories, each group was described using a specific set of equations. Model calibration was performed independently for each animal category using published experimental data and reference values from the Nitrates Directive (91/676/EEC). On-farm data collection was therefore focused on targeted sampling of faeces and urine from lactating cows, which represent the animal category contributing most to overall manure output.

**Figure 1:**
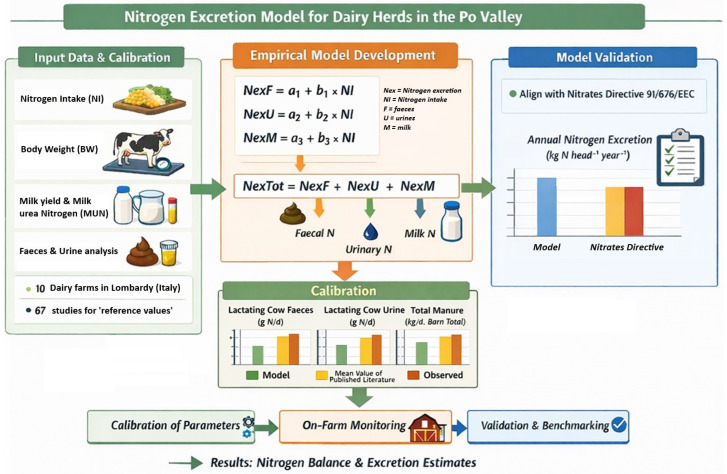
Schematic representation of the farm-scale nitrogen excretion modelling framework, including input data acquisition, empirical model development, parameter calibration using on-farm and literature data, and validation against Nitrates Directive excretion factors, leading to nitrogen balance and excretion estimates.

The Materials and Methods section describes the development, calibration and evaluation of this integrated farm-scale modelling framework aimed at estimating nitrogen excretion under commercial dairy production conditions, without inferential or hypothesis-testing objectives. A visual representation of the modelling workflow is shown in Figure 1**.**

### 2.1. Model Definition

Model development began with the identification of empirical prediction equations from peer-reviewed literature describing the relationships between nitrogen intake (NI) and nitrogen excretion pathways for each animal category. Candidate equations were selected based on three criteria: (i) their relevance to production systems comparable to those of the Po Valley (Italy) characterised by Holstein cows managed under TMR feeding, (ii) the use of predictors routinely measured on commercial dairy farms, and (iii) the extent of validation reported in the original studies. When multiple alternative equations were available for the same response variable, a single equation was selected in order to maintain model simplicity and internal consistency. Table 1 therefore reports only the final set of equations retained after the selection process.

The selected equations were integrated into a coherent modelling framework representing nitrogen flows within the barn system. In this context, the term *model* refers to an organised system of interrelated equations that allows the consistent estimation of faecal, urinary and, for lactating cows, milk nitrogen excretion across animal categories. The resulting model is empirical in nature, relying on linear or nonlinear relationships linking NI to nitrogen excretion pathways, with functional forms varying according to animal category. Hereafter, the term ‘model’ is used to refer to this integrated set of equations. Although NI constituted the primary predictor in all equations, additional variables were incorporated when required by the selected empirical equations. These included body weight (BW) for estimating urinary N in non-lactating animals; dry matter intake (DMI) for deriving total manure output; milk yield and milk protein content for calculating N secretion in milk; and milk urea nitrogen (MUN) for predicting urinary N in lactating cows. Diet composition (crude protein, N content, fibre fractions) was used exclusively to compute NI and was not included as an independent predictor in excretion equations, in line with the objective of developing a simplified tool suitable for farm-level application.

Using these literature-based equations as a foundation, a context-specific model was constructed by calibrating parameter values to reflect the production conditions. Calibration was performed within the parameter variability ranges reported in the original publications, allowing preservation of the underlying empirical structure while ensuring numerical alignment with local production characteristics.

**Figure 2:**
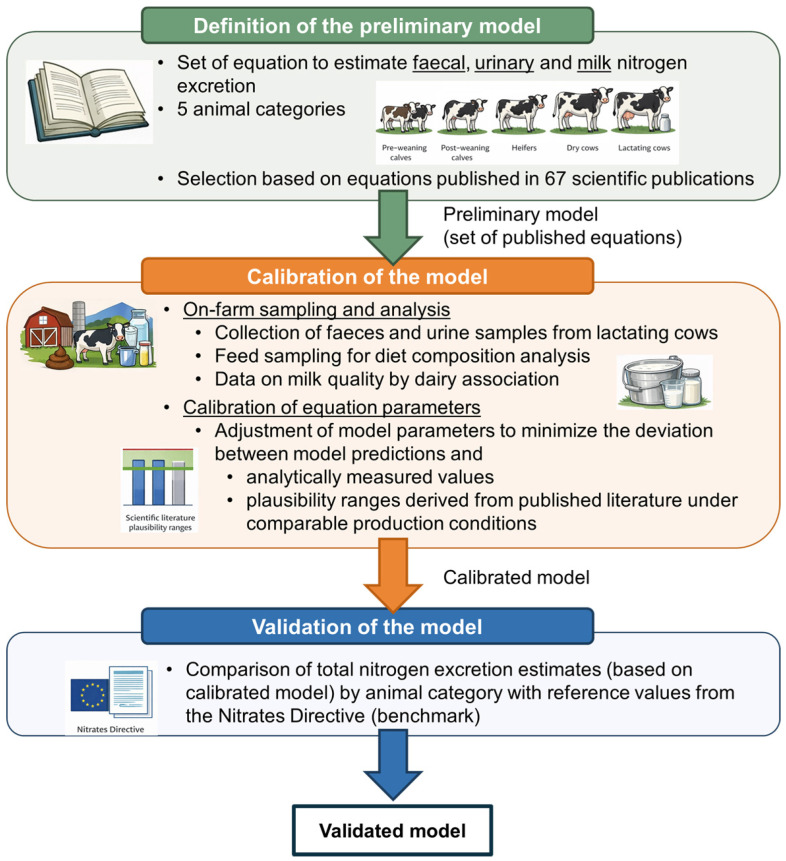
Schematic overview of the modelling approach used to estimate nitrogen excretion in dairy herds. The figure shows the definition of the preliminary model based on equations selected from the scientific literature, the calibration of model parameters using on-farm sampling data, and the validation of the calibrated model through comparison of total nitrogen excretion estimates by animal category with reference values from the Nitrates Directive and plausibility ranges reported in published studies under comparable production conditions.

All input data underwent a plausibility check prior to calibration and validation. Implausible observations, typically due to transcription errors and detected through graphical inspection of NI–Nex relationships, were removed. Missing data were replaced only when a reliable, farm-specific value was available (e.g., declared feed composition). No modelled values were subsequently removed after calibration. A schematic overview of the modelling framework and the relationships among input variables and nitrogen excretion pathways is presented in Figure 2**.**

### 2.2. Farm Monitoring and Data Collection

The calibration dataset was generated from ten commercial Holstein dairy farms located in Lombardy and monitored over a 25-month period (June 2021–June 2023). Farms were intentionally selected to capture the structural, nutritional and management variability that characterises dairy production systems in the Po Valley, where Holstein cows represent the predominant dairy breed. Although the number of farms was limited by project-specific constraints, a purposive and stratified sampling approach was adopted. In particular, farms were selected across three herd-size classes based on the number of lactating cows and according to the level of technological adoption, including both technologically advanced farms (e.g., equipped with milking robots and/or anaerobic digesters) and more traditional production systems. Farm selection was carried out in collaboration with experts from the dairy sector to ensure representativeness of the prevailing production conditions in the study area. Across farms, the average number of lactating cows was 217 ± 193, with a mean milk yield of 36.1 ± 3.6 kg cow^−1^ d^−1^.

Diet composition for all animal categories was recorded quarterly. Feed ingredients were sampled annually in accordance with Commission Regulation (EU) No. 691/2013 [31], immediately frozen at −20 °C, and subsequently analysed for dry matter and crude protein using AOAC method 2001.11 [32]. Ration composition and nutrient profiles were determined using Razio-Best software (version 5.60), which applies the Cornell Net Carbohydrate and Protein System (CNCPS) for nutrient estimation.

Milk yield and composition (fat, protein, casein, lactose), parity, days in milk, and milk urea nitrogen (MUN) were supplied quarterly by the Italian Breeders Association (AIA) at the individual animal level. For the calibration of faecal and urinary N excretion in lactating cows, twenty cows per farm (DIM > 90 d) were sampled each quarter. Faeces were collected immediately after spontaneous defecation, homogenised, and frozen at −20 °C until analysis. Urine was collected during spontaneous urination, ensuring minimal contamination, and stored under identical conditions. Both matrices were analysed for dry matter and crude protein following the same laboratory protocols applied to feed samples, ensuring analytical consistency across input variables.

Only non-invasive sampling procedures were employed, involving spontaneous excreta collection without restraint, handling stress or experimental manipulation. No interventions capable of inducing pain or discomfort were performed; therefore, approval from an Institutional Animal Care and Use Committee (IACUC) was not required under Italian legislation.

### 2.3. Model Calibration

Model calibration was carried out by adjusting the parameters of the preliminary model so that estimated nitrogen excretion values were consistent with available empirical and literature-based reference data (Appendix A, Table A1). As a first step, the preliminary model was used to estimate daily nitrogen excretion in faeces, urine and, only for lactating cows, milk.

For lactating cows, model estimates of faecal and urinary nitrogen excretion were compared with analytically determined values obtained from on-farm sampling. Observed daily excretion was calculated by combining measured nitrogen concentrations in faeces and urine with predicted faecal and urinary output. These analytically derived values were used as reference targets for calibration.

For pre-weaning calves, post-weaning calves, heifers and dry cows, no direct on-farm measurements were available. Therefore, model estimates for these categories were evaluated against plausibility ranges derived from a compilation of 67 peer-reviewed studies reporting individual animal nitrogen excretion data under comparable production conditions.

As an additional consistency check at farm scale, total manure nitrogen measured analytically at barn level—including excreta from all animal categories—was compared with the corresponding modelled estimates obtained by aggregating category-specific outputs.

Parameter calibration was performed through a least-squares minimisation procedure, reducing the deviation between model estimates and observed or literature-based reference values. Parameter adjustments were constrained within the variability ranges reported in the original equations to preserve biological plausibility. This calibration strategy follows commonly adopted recommendations for the evaluation and refinement of empirical nutritional models, ensuring that published relationships are adapted to the production context of Holstein cows managed with TMR in the Po Valley. This iterative process resulted in the final calibrated version of the model.

### 2.4. Model Validation

Model validation focused on assessing the agreement between predictions generated by the calibrated model and the nitrogen excretion factors adopted by the Lombardy Region for implementing the Nitrates Directive (91/676/EEC). Because the Directive expresses nitrogen excretion after accounting for storage-related losses, the regional excretion factors were adjusted by +28% to approximate gross nitrogen excretion at barn level, corresponding to the system boundary of the present model.

Annual nitrogen excretion predicted for each animal category (kg N head^−1^ year^−1^) was compared directly with these adjusted regulatory reference values. This comparison served to evaluate the external consistency of the model and its alignment with the official framework used in Lombardy for regulatory reporting, compliance with the Nitrates Directive, and manure management planning. By benchmarking model outputs against regionally adopted excretion factors, the validation procedure assessed whether the simplified empirical model can be reliably integrated into existing environmental accounting and farm advisory tools.

All analyses were performed in R version 4.3.1.

## 3. Results

During calibration, nitrogen excretion was estimated separately for each animal category, and model parameters were optimised using an error minimisation approach to reduce discrepancies between predicted values and laboratory-measured nitrogen excretion in faeces, urine, milk, and manure (lactating cows), or literature-based biological and statistical ranges for the other animal categories (see Appendix A, Table A2 and Table A3). During this process, the parameters of the prediction equations were adjusted to ensure consistency with observed variability while preserving the empirical structure of the equations.

Upon completion of the calibration, a simplified herd-level nitrogen excretion model adapted to the dairy production conditions of the Po Valley (Italy) was obtained, and the final calibrated parameters are reported in Table 2.

The following section presents the nitrogen excretion estimates generated by the calibrated model for each animal category. These estimates are compared with the nitrogen excretion factors established under the Nitrates Directive as part of the model validation.

### 3.1. Pre-Weaning Calves

The diet provided to pre-weaning calves, consisting of cow milk or milk replacer, was characterised by low fibre and high digestibility. Nitrogen intake (NI) averaged 37.9 g/d (Table 3), falling below the 47.0 g/d requirement reported by NASEM [33] for calves of similar body weight (65 kg). The calibrated model predicted faecal and urinary nitrogen excretion of approximately 3.0 g/d and 16.6 g/d, respectively. Annual N excretion estimated by the model was consistent with both the Nitrates Directive reference value (7.1 kg/year) and published literature, which reports comparable means around 7.5 kg/year [34,35,36,37,38], confirming the adequacy of the model for this animal category (Table 4).

### 3.2. Post-Weaning Calves

Post-weaning calves showed a mean nitrogen intake (NI) of 94.4 g/d, reflecting the transition to more fibrous diets after weaning. These values fall within the range expected for animals of comparable body weight, aligning with the nutritional requirements reported by NASEM [33] and with literature values from Lohakare et al. [39] (72–115 g/d). Compared with Kazemi-Bonchenari et al. [36], who reported NI values between 55 and 79 g/d for calves aged 53–73 days, modelled NI values were slightly higher, as expected due to the older age and larger body weight of the animals included in the present study.

The calibrated model predicted faecal N excretion (NexF) at 27.8 g/d and urinary N excretion (NexU) at 43.5 g/d (Table 3). These estimates are consistent with the benchmark values reported by Nennich et al. [27], who predicted NexF and NexU values of 23.6 g/d and 39.3 g/d, respectively, confirming the biological plausibility of the model outputs.

Annual nitrogen excretion estimated by the model averaged 26.0 kg/year, showing close alignment with both the Nitrates Directive reference value (23.96 kg/year) and published literature (24.31 kg/year). This agreement across multiple external datasets suggests that the calibrated model provides estimates for post-weaning calves that are consistent with published reference values (Table 4).

### 3.3. Heifers

Heifers showed an average nitrogen intake (NI) of 155.6 g/d, within a range of 79.9–210.5 g/d. The dietary crude protein (CP) content averaged 12.56% DM, consistent with NASEM [33] recommendations for heifers of 330–420 kg BW (CP: 11.7–12.6% DM).

The calibrated model estimated faecal N excretion (NexF) at 48.4 g/d and urinary N excretion (NexU) at 87.0 g/d, confirming urine as the primary excretion route (Table 3). These trends are consistent with previous findings: Gabler and Heinrichs [40] reported increasing total N excretion at higher dietary CP levels, while Marini and Van Amburgh [41] observed that increasing CP did not affect faecal N but led to linear increases in urinary N, N digestibility and total N excretion. The model reproduced this biological pattern, with rising dietary N leading to increased NexU and urea-N representing the majority of the additional urinary loss.

Model predictions also aligned with established relationships between N supply and N utilisation efficiency, with improved efficiency at lower N intakes as reported by Marini and Van Amburgh [41].

Annual N excretion predicted for heifers averaged 49.9 kg/year, in close agreement with published values (44.4 ± 3.74 kg/head/year). In contrast, the Nitrates Directive reports a substantially higher mean of 57.0 kg/year, suggesting a likely overestimation in the Directive’s factor or a conservative regulatory assumption. The proximity of model predictions to experimental data supports the reliability of the calibrated model for this animal category (Table 4).

### 3.4. Dry Cows

Dry cows showed an average nitrogen intake (NI) of 219.3 g/d (range: 144.6–311.9 g/d; Table 3), consistent with nutritional requirements for this physiological stage [33]. The calibrated model predicted faecal N excretion (NexF) of 68.3 g/d and urinary N excretion (NexU) of 118.5 g/d, resulting in a total N excretion (NexTot) of 186.8 g/d. Nitrogen balance (NB) was positive (mean: 32.5 g/d), reflecting body N retention, in agreement with previous studies [42,43,44].

The predicted faecal and urinary excretion values were consistent with published estimates for dry cows [27,29,44], further supporting the biological plausibility of the model outputs.

On an annual basis, the model estimated a mean N excretion of 68.2 kg/year, closely matching published values (54.7 kg/year). In contrast, the Nitrates Directive reports a substantially higher value (119.4 kg/year). The marked discrepancy between the Directive’s figure and both the model predictions and literature suggests a potential overestimation in the regulatory excretion factor, or alternatively, a conservative assumption intended for regulatory purposes.

### 3.5. Lactating Cows

Sampled lactating cows averaged 181 days in milk (DIM) and had a mean parity of 2.07 (range: 1.74–2.39). The average daily milk yield was 36.1 kg/d (30.4–42.7 kg/d), while milk composition was characterised by 3.83% fat (2.35–4.60%) and 3.38% protein (3.10–3.66%). Milk urea nitrogen (MUN) concentrations averaged 24.28 mg/dL (15.54–34.91 mg/dL), values consistent with high-producing herds [45]. Nitrogen intake (NI) averaged 598.8 g/d (range: 425.6–787.8 g/d; Table 3), with an average N content of 2.45% DM in alignment with NASEM [33] recommendations.

Measured faecal nitrogen excretion averaged 185.2 g/d, closely matching the model prediction (180.1 g/day). Urinary nitrogen excretion also showed strong agreement between measured and predicted values (176 and 179.9 g/d, respectively). Milk nitrogen excretion followed the same pattern, with measured and predicted values of 194.8 and 204.8 g/d, respectively (Table 3). These results fall well within the range reported in previous studies and confirm the biological coherence of the model (Table 4).

Nitrogen balance exhibited the expected variability for lactating cows, but the mean measured value (42.4 g/d) and predicted value (34.0 g/d) remained comparable and consistent with published patterns of nitrogen retention and mobilisation.

On an annual basis, modelled nitrogen excretion averaged 132.5 kg/year, closely matching both the predicted reference value (131.5 kg/year) and literature estimates (131.2 kg/year). The Nitrates Directive provides a slightly higher estimate (152.9 kg/year), though still within a similar magnitude. Overall, the comparison among measured data, model predictions and published values shows a satisfactory agreement for lactating cows (Table 4).

### 3.6. Model Exploitation and Limitations

The results of this study demonstrate that the proposed herd-level nitrogen model represents a practical, calibrated and context-specific tool for estimating nitrogen excretion in dairy farms of the Po Valley. Its simplicity, stemming from the use of routinely collected farm data, combined with system-level calibration based on regional production conditions, gives the model considerable potential both for on-farm decision making and for broader territorial planning. The proposed model can be usefully applied in dairy farms to routinely assess their environmental performance over time and to derive farm-specific excretion factors, which can be directly integrated into the documentation required under the Nitrates Directive. Moreover, continuous monitoring allows the identification of deviations from reference excretion values, supporting the investigation of underlying management or feeding issues and the definition of targeted mitigation strategies.

The added value of this work lies in the development of a tool that bridges a persistent gap between complex research-oriented models and the need for easy-to-implement, locally calibrated instruments. Unlike generic excretion factors or mechanistic models with high data demands, the present model provides estimates that are biologically coherent, regionally relevant and directly applicable in commercial settings. It therefore represents a scalable resource for environmental reporting, manure management planning and potential policy design in nitrate-vulnerable zones.

From a methodological standpoint, the use of readily available farm-level data—such as ration composition, milk records and basic animal characteristics—substantially increases the likelihood of adoption by farmers, consultants and researchers. This aligns with previous evidence showing that tools grounded in accessible datasets are more easily integrated into decision-making processes [25,26].

A limitation of the study is the restricted number of farms included in the calibration dataset. However, this constraint is partially mitigated by the longitudinal structure of the data, which comprise repeated measurements collected over a 25-month monitoring period and a large number of individual animals sampled per farm. This temporal coverage helps to reduce the influence of seasonal, managerial and nutritional variability and enhances the representativeness of the calibration within the production context considered.

Direct biological measurements were available only for lactating cows, as this category represents the main contributor to farm total nitrogen excretion and plays a central role in on-farm feeding management. For pre-weaning calves, post-weaning calves, heifers and dry cows, nitrogen excretion estimates relied exclusively on published literature data. Consequently, model performance for these categories was assessed through descriptive comparison with literature-derived plausibility ranges rather than independent biological verification, and this aspect should be considered when interpreting the results.

Moreover, on-farm sampling was based on direct collection of faeces and urine, which is inherently less precise than marker-based methodologies. Nevertheless, model outputs for lactating cows were consistent with published values obtained using more controlled analytical approaches, and the comparison with excretion factors adopted for regulatory implementation provided an additional descriptive benchmark at farm scale.

Finally, aspects related to the quantitative characterisation of variability and uncertainty were not explicitly addressed within the scope of the present study. This work should therefore be regarded as a first step toward the development of an integrated farm-scale framework for nitrogen excretion assessment. Future studies should focus on expanding experimental validation across animal categories, incorporating explicit uncertainty and sensitivity analyses, and testing the framework under a wider range of production and management conditions to further characterise its applicability.

## 4. Conclusions

This study developed and calibrated a simplified herd-level model for estimating nitrogen excretion in dairy farms under the production conditions of the Po Valley. By relying on nitrogen intake as the principal driver and on predictors routinely available in commercial farm records, the model provides biologically consistent and locally calibrated estimates of faecal, urinary and milk nitrogen excretion across all animal categories. The close agreement between model predictions, measured values and published data supports the consistency oof the modelling framework, while the comparison with the excretion factors used for the Nitrates Directive highlights its potential as a complementary or alternative tool for regional N accounting.

A major contribution of this work lies in the development of a practical and scalable decision-support instrument that bridges the gap between complex research-oriented models and the need for simple, operational tools at farm level. Because the model is directly applicable at herd scale using readily available data, it enables farms to routinely evaluate their nitrogen management performance over time and to derive herd-specific nitrogen excretion coefficients. This supports the optimisation of feeding strategies aimed at improving nitrogen use efficiency while mitigating environmental impacts. At the same time, the regional calibration of the model enhances its relevance for territorial planning and policy development, particularly in nitrate-vulnerable areas, where reliable and farm-specific estimates of nitrogen excretion are essential for regulatory compliance, environmental monitoring, and the identification of targeted mitigation actions.

Although the number of monitored farms was limited, the dataset is strengthened by the large number of animals sampled and by repeated measurements collected over a 25-month period, enhancing the representativeness and stability of the calibration. Future research could expand direct measurements to additional animal categories, integrate dynamic dietary changes, or explore the incorporation of marker-based techniques to further refine model accuracy.

Overall, the proposed model represents a meaningful advancement toward bridging scientific knowledge and practical implementation, offering an accessible tool for improving nitrogen management at both farm and regional levels.

## Figures and Tables

**Table 1:** Selected Equations for Nitrogen Excretion by Animal Category.

Category	Units	Equations	Ref.
***Lactating cows***			
NexF	(g/d)	$35.4\;\left(\pm 3.15\right)+0.25\;\left(\pm 0.004\right)\times NI$	[25]
NexU	(g/d)	20.2 (±5.65)+0.28 (±0.008)×NI	[25]
NexM	(g/d)	97.3 (±5.44)+0.096 (±0.008)×NI	[26]
Total manure	(kg/d)	2.63 (±0.10)+9.4 (±2.8)×DMI	[27]
Urine	(kg/d)	0.563 (±0.115)+17.1 (±2.0)×MUN	[28]
***Dry cows and Heifers***			
NexU	(g/d)	14.3 (±3.8)+0.510 (±0.0121)×NI	[29]
NexF	(g/d)	0.345 (±1.73)+0.317 (±0.00638)×NI	[29]
Total manure	(kg/d)	4.158×DMI−0.0246×BW	[27]
***Pre-weaned calves***			
NexU	(g/d BW^0.75^)	$0.278\;\left(\pm 0.025\right)+1.146\;\left(\pm 0.128\right)\times NI\times e^{-3\;\times \;\frac{\left[0.698(\pm 0.097)\right]}{NI}}$	[30]
NexF	(g/d DMI)	3.05 (±0.40)−0.004 (±0.009)×NI	[30]
Total manure	(kg/d)	DMI×3.45 (±0.041)	[27]
***Post-weaned calves***			
NexU	(g/d BW^0.75^)	$0.123\;\left(\pm 0.036\right)+1.146\;\left(\pm 0.128\right)\times NI\times e^{-3\;\times \;\frac{\left[0.698(\pm 0.097)\right]}{NI}}$	[30]
NexF	(g/d DMI)	$6.51\;\left(\pm 0.31\right)+0.036\;\left(\pm 0.011\right)\times NI$	[30]
Total manure	(kg/d)	3.45 (±0.041)×DMI	[27]

BW = Body weight; NI = Nitrogen Intake; DMI = Dry Matter Intake; NexF = Faecal N excretion; NexU = Urine N excretion; NexM = Milk N excretion; MUN = Milk urea nitrogen.

**Table 2:** Simplified Herd Model for Nitrogen Excretion.

Category	Units	Equations	References
*Lactating cows*			
NexF	(g/d)	33.4 + 0.245 × NI	***Calibrated*** [25]
NexU	(g/d)	15.2 + 0.275 × NI	***Calibrated*** [25]
NexM	(g/d)	97.3 + 0.096 × NI	[26]
Total manure	(kg/d)	9.4 + 2.63 × DMI	[27]
Urine	(kg/d)	0.56 + 17.1 × MUN	[28]
*Dry cows and Heifers*			
NexU	(g/d)	11 + 0.49 × NI	***Calibrated*** [29]
NexF	(g/d)	0.345 + 0.31 × NI	***Calibrated*** [29]
Total manure	(kg/d)	4.158 × DMI − 0.0246 × BW	[27]
*Post-weaned calves*			
NexU	(g/d BW^0.75^)	0.115 + 1 × NI × $e^{-3\;\times \;\frac{0.698}{NI}}$	***Calibrated*** [30]
NexF	(g/d DMI)	6.3 + 0.03 × NI	***Calibrated*** [30]
Total manure	(kg/d)	3.45 × DMI	[27]
*Pre-weaned calves*			
NexU	(g/d BW^0.75^)	0.255 + 1.12 × NI $\times \;e^{-3\;\times \;\frac{0.698}{NI}}$	***Calibrated*** [30]
NexF	(g/d DMI)	3.4 − 0.001 × NI	***Calibrated*** [30]
Total manure	(kg/d)	3.45 × DMI	[27]

BW = body weight; NI = Nitrogen Intake; DMI = Dry Matter Intake; NexF = Fecal N excretion; NexU = Urine N excretion; NexM = Milk N excretion; MUN = Milk urea nitrogen.

**Table 3:** Summary descriptive statistics of number of animals, body weight, nitrogen intake, and nitrogen excretion (estimated by model or by lab analyses) by animal category.

	Pre-Weaning Calves		Post-Weaning Calves		Heifers		Dry Cows		Lactating Cows			
	Mean ± SD	[Min; Max]	Mean ± SD	[Min; Max]	Mean ± SD	[Min; Max]	Mean ± SD	[Min; Max]	Mean ± SD	[Min; Max]	Mean ± SD	[Min; Max]
Number of animals (n)	43.1 ± 43.5	[6; 139]	60.6 ± 51.1	[8.0; 148]	178 ± 160	[22.0; 531]	39.7 ± 28.4	[8.0; 91.0]	217 ± 193	[35; 601]		
BW (kg)	80 ± 0	[80; 80]	140 ± 0	[140; 140]	334 ± 22.8	[315; 410]	688 ± 9.9	[680; 700]	688 ± 9.9	[680; 700]		
	Model-based estimates		Model-based estimates		Model-based estimates		Model-based estimates		Model-based estimates		Analytically determined	
NI (g/d)	37.9 ± 2.4	[33.6; 41.6]	94.4 ± 17.7	[67.4; 136]	156 ± 33.5	[79.9; 210]	219.3 ± 39.4	[145; 312]	599 ± 77.8	[426; 788]		
NexF (g/d)	2.9 ± 0.2	[2.5; 3.3]	27.8 ± 6.8	[16.3; 37.7]	48.4 ± 10.5	[25.1; 65.6]	68.3 ± 12.2	[45.2; 97.0]	180.1 ± 19.1	[138; 226]	185 ± 43.0	[74.2; 277]
NexU (g/d)	16.6 ± 1.5	[13.9; 18.9]	43.5 ± 14.0	[23.7; 77.6]	87.0 ± 16.6	[50.1; 114]	118 ± 19.31	[81.9; 164]	179.9 ± 21.4	[132; 232]	176 ± 43.9	[90.7; 276]
NexM (g/d)									205 ± 7.5	[188; 223]	195 ± 17.7	[162; 229]
NexTot (g/d)	19.5 ± 1.6	[16.9; 21.9]	71.3 ± 19.2	[40.1; 112]	135 ± 27.1	[75.2; 179]	186.8 ± 31.5	[127; 261]	567 ± 48.0	[458; 681]	556 ± 57.5	[450; 689]
NB (g/d)	18.4 ± 0.8	[16.7; 19.7]	23.1 ± 5.1	[12.2; 30.3]	19.68 ± 6.78	[4.6; 30.8]	32.5 ± 7.9	[17.6; 51.0]	34.0 ± 29.9	[−32.5; 107]	42.4 ± 72.4	[−158; 157]
Total Faeces and Urine (kg/d)	3.0 ± 0.2	[2.5; 3.3]	13.7 ± 3.5	[7.8; 19.2]	25.05 ± 6.34	[12.0; 34.3]	36.0 ± 10.2	[20.8; 62.0]	73.7 ± 5.3	[56.8; 86.6]		
Total Faeces (kg/d)									50.2 ± 5.4	[32.5; 63.4]		
Total Urine (kg/d)									23.5 ± 1.2	[21.2; 26.3]		

NI = Nitrogen intake; NexF = Faecal N excretion; NexM = Milk N excretion; NexU = Urine N excretion; NexTot = Total N excretion; NB = N balance (NI-NexTot). Data on body weight (BW) were collected through the Agronomic Use Plan for Livestock Effluents (PUA).

**Table 4:** Summary descriptive statistics of NexF and NexU (kg/year) for measured, predicted, Nitrates Directive, and published values across different livestock categories.

	Measured Values		Predicted Values		Nitrates Directive		Published Values	
Category	Mean	SD	Mean	SD	Mean	SD	Mean	SD
Pre-weaning calves	-	-	7.11	0.59	7.11	0.00	7.49	0.00
Post-weaning calves	-	-	26.02	6.97	24.31	0.00	23.96	0.00
Heifers	-	-	49.92	3.74	57.03	14.56	44.43	3.74
Dry cows	-	-	68.19	9.74	119.44	1.79	54.74	0.00
Lactating cows	132.51	13.42	131.48	12.79	152.89 ^1^ 131.87 ^2^	2.30 ^1^ 1.98 ^2^	131.19	0.00

^1^ Dairy cows with live weight of 600 kg/head, milk yield of 35 kg milk/d, and optimal feeding (Nitrates Directive, European Directive 91/676/EC). ^2^ Dairy cows with live weight of 600 kg/head, milk yield of 35 kg milk/d, and normal feeding (Nitrates Directive, European Directive 91/676/EC). -: no data available for this category.

## Data Availability

The data that support the findings of this study are available from the corresponding author upon reasonable request.

## Abbreviations

The following abbreviations are used in this manuscript:

AIA	Associazione Italiana Allevatori—Italian Breeders Association
BW	Body weight
CNCPS	Cornell Net Carbohydrate and Protein System
DIM	Days in milk
DMI	Dry Matter Intake
IACUC	Institutional Animal Care and Use Committee
MUN	Milk urea nitrogen
NB	Nitrogen balance
NI	Nitrogen intake
NexF	Nitrogen excretion in faeces
NexM	Nitrogen excretion in milk
NexTot	Total nitrogen excretion
NexU	Nitrogen excretion in urines
TMR	Total Mixed Rations

## Appendix A

**Table A1:** Selected bibliography for the input variables and/or references used in the model calibration.

Input Variable	References
***Pre-weaning calves***	
NI (g/d)	[34,35,36,37,46]
Dietary nitrogen content (%)	[34,35,46]
NexF (g/d)	[34,35,38,47]
NexU (g/d)	[34,35,37,38,47]
***Post-weaning calves***	
NI (g/d)	[36,39,48]
Dietary nitrogen content (%)	[36]
NexF (g/d)	[39,48]
NexU (g/d)	[39]
***Heifers (13–24 months)***	
NI (g/d)	[29,49,50,51,52]
Dietary nitrogen content (%)	[49]
NexF (g/d)	[29,49,50,51,52]
NexU (g/d)	[29,49,50,51,52,53]
NexTot (g/d)	[29,52]
***Heifers (7–12 months)***	
NI (g/d)	[40,41,49,54,55]
N (%)	[49]
NexF (g/d)	[40,41,49,54,55]
NexU (g/d)	[40,41,49,54,55]
***Dry cows***	
NI (g/d)	[29,42]
DMI (kg/d)	[29,42,44]
Dietary nitrogen content (% DM)	[29,42,44]
NexF (g/d)	[29,42,44]
NexU (g/d)	[29,42,44]
NexTot (g/d)	[29,42]
***Lactating cows***	
Body weight (kg)	[25,29,56,57,58,59]
Dietary nitrogen content (% DM)	[25,56,57,58,60,61,62,63,64,65]
DMI (kg/d)	[25,57,61,62,65]
Faecal dry matter content (%)	[66,67,68,69]
Faecal nitrogen content (% DM)	[59,66,67,68,69,70,71,72,73,74]
Milk fat (%)	[25,57,58,59]
Milk protein (%)	[57]
MUN (mg N/dL)	[25,57,58,59,75]
Milk yield (kg/d)	[25,56,57,58,59]
NexF (g/d)	[25,57,63,64,67,76,77,78,79,80,81,82,83,84,85,86,87]
NexM (g/d)	[57,59,60,63,64]
NexU (g/d)	[25,63,64,67,75,76,77,78,79,80,81,82,83,84,86,87]
NexTot (g/d)	[3,25,29]
NI (g/d)	[25,57,60,61,65]
Urine nitrogen content (%)	[67,68,73,75,85,88,89,90,91,92,93,94]

DMI: Dry matter intake; MUN: Milk urea nitrogen; NexF: Nitrogen excretion in faeces; NexM: Nitrogen excretion in milk; NexTot: Total nitrogen excretion; NexU: Nitrogen excretion in urine; NI: Nitrogen intake; Dietary nitrogen content.

**Table A2:** Summary of animal and dietary characteristics, faecal and urinary traits, and nitrogen excretion in lactating and dry dairy cows from the published literature.

	*Lactating Cows*			*Dry Cows*		
*Input Variable*	*n*	*Mean*	*SD*	*n*	*Mean*	*SD*
***Animal characteristics***						
DMI (kg/d)	***6071***	***21.65***	***6.64***	***1450***	***6.05***	***4.47***
BW (kg)	***6089***	***633.47***	***59.79***	***-***	***-***	***-***
***Dietary characteristics***						
NI (g/d)	***6156***	***589.38***	***320.35***	***1442***	***148.72***	***46.17***
N (% DM)	***6597***	***2.69***	***0.92***	***1446***	***2.50***	***0.37***
***Faecal and urine characteristics***						
Faecal dry matter content DM (%)	***59***	***15.50***	***11.25***	***-***	***-***	***-***
Faecal nitrogen content N (% DM)	***299***	***2.46***	***0.72***	***24***	***0.22***	***0.01***
Urine nitrogen content N (%)	***189***	***0.88***	***0.48***			
***Nitrogen excretion***						
NexF (g/d)	***5464***	***183.54***	***159.14***	***1450***	***49.97***	***28.03***
NexU (g/d)	***3818***	***175.89***	***165.08***	***986***	***95.53***	***44.81***
NexM (g/d)	***695***	***165.14***	***92.52***	***-***	***-***	***-***
NexTot (g/d)	***5221***	***370.42***	***240.81***	***1426***	***131.13***	***47.81***
***Productive performance***						
MY (kg/d)	***5610***	***32.45***	***12.41***	***-***	***-***	***-***
MFat (%)	***5004***	***3.88***	***0.78***	***-***	***-***	***-***
Mprot (%)	***200***	***3.16***	***0.19***	***-***	***-***	***-***
MUN (mg N/dL)	***4664***	***11.03***	***2.98***	***-***	***-***	***-***

**Table A3:** Summary of animal and dietary characteristics, faecal and urinary traits, and nitrogen excretion in pre and post-weaning calves and in Heifers from published studies.

	Pre-Weaning Calves			Pos-Weaning Calves			Heifers (7–12 Months)			Heifers (13–24 Months)		
Variable	n	Mean	SD	n	Mean	SD	n	Mean	SD	n	Mean	SD
*Dietary characteristics*												
NI (g/d)	136	36.30	48.39	54	96.28	157.40	70	161.56	192.04	1479	149.06	87.85
*Faecal and urine characteristics*												
Faecal N (%)	109	4.26	2.99	-	-	-	-	-	-	-	-	-
Urine N (%)	72	0.16	0.60	106	0.23	0.06	6	1.15	0.54	6	1.03	0.61
*Nitrogen excretion*												
NexF (g/d)	291	6.87	17.71	54	38.21	28.96	6	45.55	12.29	1479	49.56	24.25
NexU (g/d)	303	13.66	13.54	36	27.43	13.03	22	66.20	170.61	1434	82.14	96.16
